# Expression Profiles of CircRNA and mRNA in Lacrimal Glands of AQP5^–/–^ Mice With Primary Dry Eye

**DOI:** 10.3389/fphys.2020.01010

**Published:** 2020-09-03

**Authors:** Yaning Liu, Guohu Di, Shaohua Hu, Tianyu Zhao, Xinkai Xu, Xiaoyi Wang, Peng Chen

**Affiliations:** Department of Human Anatomy, Histology and Embryology, School of Basic Medicine, Qingdao University, Qingdao, China

**Keywords:** AQP5, lacrimal gland, dry eye, circRNA, miRNA, mRNA

## Abstract

**Purpose:** This work aimed to identify differentially expressed circular RNAs (circRNAs) and elucidate their potential function in aquaporin 5 (AQP5) knockout (AQP5^–/–^) mice with the primary dry eye phenotype.

**Methods:** A slit lamp examination was performed on AQP5^–/–^ mice to assess corneal epithelial defects using fluorescein sodium staining. Hematoxylin–eosin staining and transmission electron microscopy analysis were performed to identify structural changes in lacrimal gland epithelial cells due to AQP5 deficiency. The expression profiles of circRNA and messenger RNA (mRNA) were determined by a microarray analysis. The selected circRNA was verified by quantitative real-time reverse transcription-polymerase chain reaction (qRT-PCR). Gene Ontology and Kyoto Encyclopedia of Genes and Genomes (KEGG) pathway enrichment analyses were performed to predict the biological functions and the potential pathways of parental genes involved in lacrimal gland epithelial cell changes. According to the bioinformatics analysis of identified circRNAs, we predicted a circRNA–miRNA–mRNA network of phagosomes.

**Results:** The AQP5^–/–^ mice spontaneously exhibit dry eye symptoms, wherein the AQP5 deficiency changes the structure of lacrimal gland epithelial cells. The analysis revealed that, compared to AQP5^+/+^ mice, 30 circRNAs in the lacrimal glands of AQP5^–/–^ mice were differentially expressed (fold change ≥ 2.0, *p* < 0.05). Nine upregulated circRNAs were identified using qRT-PCR, and nine upregulated validated circRNAs, 40 altered microRNAs (miRNAs), and nine upregulated mRNAs were identified through a network analysis. The KEGG analysis showed that these nine target genes were expressed in phagosomes.

**Conclusion:** The AQP5^–/–^ mice have primary and stable dry eye phenotypes from birth. We identified differently expressed circRNAs in the lacrimal glands of AQP5^–/–^ and AQP5^+/+^ mice, predicting a circRNA–miRNA–mRNA network of phagosomes. CircRNA likely plays an important role in lacrimal gland epithelial cell pathogenesis. Therefore, it is reasonable to use circRNA as a potential therapeutic agent for the treatment of dry eyes.

## Introduction

Dry eye is currently the most common eye disease except for ametropia, with a worldwide incidence of 5–34% (Messmer, 2015). The main pathophysiological mechanisms are tear film instability, increased tear permeability, ocular surface inflammation and injury, and neurosensory abnormalities (Craig et al., 2017). The current treatment strategies for dry eye include the use of artificial tears, administration of anti-inflammatory drugs, and surgery (Lemp and Foulks, 2007). However, the incidence of dry eye is still rising, emphasizing the urgent need to determine its pathogenesis and to develop effective treatment measures.

Aquaporin 5 (AQP5), a kind of aquaporin, is highly expressed in the corneal epithelium and lacrimal glands and is closely related to eye diseases (Raina et al., 1995; Takata et al., 2004). Compared with AQP5^+/+^ mice, the lenses of AQP5^–/–^ mice *in vitro* appear to be slightly turbid at high glucose concentrations, suggesting that AQP5 might maintain crystal transparency by regulating osmotic pressure (Sindhu Kumari and Varadaraj, 2013). AQP5 has also been shown to reduce saliva and airway mucus secretions and increase corneal thickness in experimental animals (Ma et al., 1999; Thiagarajah and Verkman, 2002). In addition, lack of AQP5 affects the migration and the proliferation of cells, leading to slow corneal healing (Kumari et al., 2018). One of the symptoms of dry eye is an epithelial defect in the cornea (Levin and Verkman, 2006). A previous study considered AQP5 level as an index of dry eye (Lin et al., 2019), and the occurrence of dry eye is believed to be accompanied by a decrease in AQP5 level. However, whether AQP5 deficiency can cause dry eye has not been proved yet.

Circular RNA (circRNA), as a specific and specific non-coding RNA, has a closed circular structure and is more stable than long non-coding RNAs (Jiang et al., 2018). A recent study has shown that circRNA molecules are rich in microRNA (miRNA) binding sites, which play the role of an miRNA sponge in cells, thus relieving the inhibition of miRNA on its target genes and increasing the expression levels of those target genes (Kulcheski et al., 2016). CircRNA might be a biomarker for primary Sjögren’s syndrome, correlating with the primary Sjögren’s syndrome etiology such as has-circRNA-001264, has-circRNA-104121, and has-circRNA-045355 (Su et al., 2019). Meanwhile, miR-146a and miR-155 have also been reported to be related to dry eye (Shi et al., 2014). Therefore, the expression and the clinical significance of circRNA in dry eye need to be studied.

In this study, we used aquaporin 5 knockout (AQP5^–/–^) mice that exhibit dry eye characteristics and performed hematoxylin–eosin staining to determine structural changes in the lacrimal glands of these mice. We found that the lacrimal glands of AQP5^–/–^ mice exhibited abnormal changes compared with those of AQP5^+/+^ mice. The differential expression of circRNA was also found by high-throughput sequencing, and a circRNA–miRNA–mRNA network related to phagosomes was predicted. These data suggest that the lack of AQP5 may cause a differential expression of circRNA, which may, in turn, lead to the development of primary dry eye.

## Materials and Methods

### Animals

Using CRISPR/Cas9 technology, AQP5^–/–^ mice were produced by the high-flux electric transfer of fertilized eggs from Cyagen Biosciences Inc. (Guangzhou, China). We used age-matched AQP5^+/+^ and AQP5^–/–^ mice for our study. All experimental and animal care procedures were followed according to the ARVO Statement for the Use of Animals in Ophthalmic and Vision Research, and the study was approved by the Animal Care and Use Committee of Qingdao University (Qingdao, China). Spontaneous defects of the corneal epithelium were visualized by instilling 0.25% fluorescein sodium and photographing under a slit lamp (66 Vision Tech. Co., Ltd., Suzhou, China).

Tear production was examined using the phenol red thread (Jingming) test as described previously (Stevenson et al., 2014). In brief, the thread was placed on the palpebral conjunctiva of the lower eyelid at one-third of the distance from the lateral canthus for 20 s. The length of the wet portion was measured in millimeters.

### Hematoxylin–Eosin Staining and Transmission Electron Microscopy

AQP5^+/+^ and AQP5^–/–^ mice were sacrificed by cervical dislocation, and the lacrimal glands were removed for further use. The lacrimal glands were fixed in 10% buffered formalin and embedded in paraffin. Paraffin sections (4 μm) were produced using paraffin-embedded tissues. The sections were stained with hematoxylin–eosin and observed under a light microscope (Nikon Eclipse E100, Nikon, Japan). Samples of approximately 1 mm^3^ were dissected from the lacrimal glands and subsequently fixed with electron microscope fixation fluid (Servicebio, G1102), post-fixed with 1% osmic acid in 0.1 mol/L phosphate buffer, dehydrated in a graded series of ethanol, and embedded in embedding agent (SPI, 90529-77-4). The specimens were cut into 60–80 nm sections by using an ultrathin microtome (Leica UC7, Leica). The sections were double-stained with uranium and lead and observed under a transmission electron microscope (HITACHI, HT7700).

### Extraction and Separation of RNA Samples

According to the manufacturer’s instructions (Lu et al., 2018), Trizol (Invitrogen, Carlsbad, CA, United States) was used to extract RNA from the lacrimal glands of both AQP5^+/+^ and AQP5^–/–^ mice, and the optical density (OD_260__/__280_) value was determined by using a NanoDrop ND-2000 instrument (Thermo Fisher Scientific, Waltham, MA, United States) to measure the RNA concentration. RNA integrity was determined by agarose gel electrophoresis.

### High-Throughput Sequencing

High-throughput transcriptome sequencing and bioinformatics analysis were performed using Cloud-Seq Biotech (Shanghai, China). Specifically, total RNA was treated with a Ribo-Zero rRNA Removal kit (Illumina, San Diego, CA, United States) to remove ribosomal RNA (rRNA). An RNA library was constructed according to the manufacturer’s instructions using the purified RNA samples and the TruSeq Stranded Total RNA Library Prep kit (Illumina, San Diego, CA, United States). The BioAnalyzer 2100 system was used for ensuring quality control and library quantification. Then, 10-pM libraries were denatured into single-stranded DNA molecules, captured on Illumina flow cells, amplified *in situ*, and clustered, and finally, 150 cycles of sequencing were performed on the Illumina HiSeq sequencer according to the manufacturer’s instructions (Lu et al., 2018).

### CircRNA Sequencing Analysis

The paired terminal readings were obtained from the Illumina HiSeq 4000 sequencer, and quality control was performed through Q30. High-quality reads were primarily screened by Cutadapt software (version 1.9.3) (Martin, 2011), and after performing 3′ adapter trimming, low-quality reads were removed. We used STAR software (version 2.5.1b) (Dobin et al., 2013) to map and align high-quality reads with the reference genome/transcriptome. Next, we selected some nucleotide sequences from the reads as anchor points and input the results into the DCC software (version 0.4.4) (Cheng et al., 2016) that eventually compared connected and unconnected reads to identify possible circRNAs. We used EdgeR software (version 3.16.5) (Robinson et al., 2010) to normalize the data and analyze the differential expression of the identified circRNAs.

### Analysis of Differential CircRNAs and mRNAs

The differential expression of circRNAs and mRNAs between the AQP5^+/+^ and the AQP5^–/–^ mice groups was calculated using standardized readings. CircRNAs and mRNAs with a fold change of ≥2.0 and a *p* < 0.05 were considered to indicate a differential expression that was statistically significant.

### Validation of Differentially Expressed CircRNAs, miRNAs, and mRNAs

The reliability of high-throughput RNA sequencing was verified by quantitative real-time reverse transcription-polymerase chain reaction (qRT-PCR). Among all the identified differentially expressed circRNAs and miRNAs, nine upregulated circRNAs and six upregulated mRNAs were selected for validation. Glyceraldehyde-3-phosphate dehydrogenase was used as a reference for standardization. Among all the identified differentially expressed miRNAs, four downregulated miRNAs were selected for validation. U6 was used as a reference for standardization. Total RNA was reverse-transcribed into complementary DNA using the PrimeScript RT kit (Perfect Real Time; Takara, Osaka, Japan), and qRT-PCR was performed using the Applied Biosystems 7500 Fast Real-Time PCR system. Three independent experiments were performed on all samples. The expression was determined using a threshold cycle, and the relative expression level was calculated using the 2^–ΔΔCT^ method. The primers used for all the selected circRNAs are specified in Table 1. The primers for all the selected mRNAs are specified in Table 2. The primers used for miRNA reverse transcription are specified in Table 3. The primers for all the selected miRNAs used in qRT-PCR are specified in Table 4.

**TABLE 1:** The primers used in qRT-PCR.

Gene	Primer type	Primer sequence
chr5:147450653-147455188 +	Forward	CTTCGGTGCCCTCAACATCT
	Reverse	GGACTCTGTGAGACTCGCATC
chr6:143180587-143197565 +	Forward	AGGGCTTTGGCAGTGATGTC
	Reverse	TAGCAGGCGATGAGCTTGTT
chr9:3441055-3460131 +	Forward	TGGAATCTGATGAAGAATGGTCC
	Reverse	TGGCCCCCAACTTGTTCTTT
chr2:18284623-18294044-	Forward	TGACTGGCTGGAAAACAGGA
	Reverse	TCTAGCCAGTGCGTCTTCTT
chr5:90298540-90304065-	Forward	CGCTTGTTCTGCTGGGTACT
	Reverse	CAGCAGTGCCTGATAGAAGC
chr19:4342847-4394892-	Forward	GCCACTGCACAAGAAGTGTG
	Reverse	AGAAACCACTCCAGGGATGC
chr10:82492146-82492319-	Forward	CTGATTTTGCAGCCAATACACG
	Reverse	TCATTAGGGTCTGCAGTGGTT
chr14:74807474-74835775-	Forward	AGGACCAGGCAGAAGACTGT
	Reverse	GAGAGGCAGACCACACAGG
chr6:8537817-8582790 +	Forward	TCCCAGGATGGAAGTCCTTG
	Reverse	CCAGAGAAGAGGTTCGCCTT
GAPDH	Forward	AAGGTCATCCCAGAGCTGAA
	Reverse	CTGCTTCACCACCTTCTTGA

**TABLE 2:** The primers used in qRT-PCR.

Gene	Primer type	Primer sequence
5430435G22Rik	Forward	GCTGCATTCCATTCCCTGAG
	Reverse	CCCTAGTGGCCAGTCATCAA
Rilp	Forward	CAGTGAGGATGAGGATGGCT
	Reverse	TCACCCCGATACCATAAGCC
Thbs1	Forward	TCTTCCTGGCTTCCTTGAGG
	Reverse	CTGGCCAGTGTTGTCTTTCC
Ncf1	Forward	GAAGACAAAGCGAGGTTGGG
	Reverse	TTCACCTGCGTAGTTGGGAT
Msr1	Forward	GAAAAGGGAGACAGAGGGCT
	Reverse	CCAGGGAAGCCAATTTGTCC
Tubb6	Forward	CAGCAGACCAGGGAGATCTC
	Reverse	AACTCTGATGGGGTGGGAAG
GAPDH	Forward	AAGGTCATCCCAGAGCTGAA
	Reverse	CTGCTTCACCACCTTCTTGA

**TABLE 3:** The RT primers of miRNA used in reverse transcription.

Gene	Primer sequence
miR-135b-5p	GTCGTATCCAGTGCGTGTCGTGGAGTCGG CAATTGCACTGGATACGACTCACAT
miR-320-3p	GTCGTATCCAGTGCGTGTCGTGGAGTCGG CAATTGCACTGGATACGACTCGCCC
miR-135a-5p	GTCGTATCCAGTGCGTGTCGTGGAGTCG GCAATTGCACTGGATACGACTCACAT
miR-8104	GTCGTATCCAGTGCGTGTCGTGGAGTCGGCA ATTGCACTGGATACGACCGCTGC
miR-1950	GTCGTATCCAGTGCGTGTCGTGGAGTCGGCA ATTGCACTGGATACGACTGACCA
miR-3473f	GTCGTATCCAGTGCGTGTCGTGGAGTCGGC AATTGCACTGGATACGACCATCTC
miR-125a-3p	GTCGTATCCAGTGCGTGTCGTGGAGTCG GCAATTGCACTGGATACGACGGCTCC
U6	AACGCTTCACGAATTTGCGT

**TABLE 4:** The primers used in qRT-PCR.

Gene	Primer type	Primer sequence
miR-135b-5p	Forward	GGGTATGGCTTTTCATTCCT
	Reverse	CAGTGCGTGTCGTGGAGT
miR-320-3p	Forward	GGGAAAAGCTGGGTTGAGA
	Reverse	CAGTGCGTGTCGTGGAGT
miR-135a-5p	Forward	GGGTATGGCTTTTTATTCCT
	Reverse	CAGTGCGTGTCGTGGAGT
miR-8104	Forward	GGGCAGGATGAGGTGGAGT
	Reverse	CAGTGCGTGTCGTGGAGT
miR-1950	Forward	GGGTCTGCATCTAAGGATA
	Reverse	CAGTGCGTGTCGTGGAGT
miR-3473f	Forward	GGGCAAATAGGACTGGA
	Reverse	CAGTGCGTGTCGTGGAGT
miR-125a-3p	Forward	GGGACAGGTGAGGTTCTTG
	Reverse	CAGTGCGTGTCGTGGAGT
U6	Forward	CTCGCTTCGGCAGCACA
	Reverse	AACGCTTCACGAATTTGCGT

### GO and KEGG Pathway Analyses

GO and KEGG were used to analyze genes related to differentially expressed circRNAs and mRNAs. GO analysis is divided into three aspects: molecular function, biological process, and cell composition. We ranked the top 10 enriched GO terms according to the *p*-value. KEGG pathway analysis was used to analyze the possible biological functions of differentially expressed circRNA and mRNA.

### Analysis of the CircRNA–miRNA–mRNA Network and Related Prediction

The miRNA binding sites and target mRNAs were predicted using proprietary software based on TargetScan (version 7.0) and MiRanda (version 3.3a). Based on the prediction results, we used the Cycloscape software (version 3.1.0) to construct a network map of circRNA–miRNA–mRNA.

### RNA Immunoprecipitation–qPCR

RNA immunoprecipitation (RIP) analysis was performed using the EZ-Magna RIP RNA binding protein immunoprecipitation kit (Millipore, Billerica, MA, United States) and the RIP protocol. The lacrimal glands of AQP5^+/+^ mice were cleaved and incubated with RIP buffer containing magnetic beads of anti-AGO2 antibody and immunoglobulin G (IgG, Abcam) as a negative control. To analyze the enrichment of circRNAs and mRNAs, qRT-PCR was performed to analyze the extracted RNA. The primers used for all the selected circRNAs and mRNAs are specified in Tables 1, 2.

### Data Analysis

The results were expressed as mean ± standard deviation (*SD*). All the experimental data were analyzed by GraphPad Prism 7.0 (GraphPad Software Inc., La Jolla, CA, United States). A *p* < 0.05 was considered as statistically significant. The data are representative of at least three experiments.

## Results

### Dry-Eye-Like Characteristics Found in AQP5^–/–^ Mice

**FIGURE 1:**
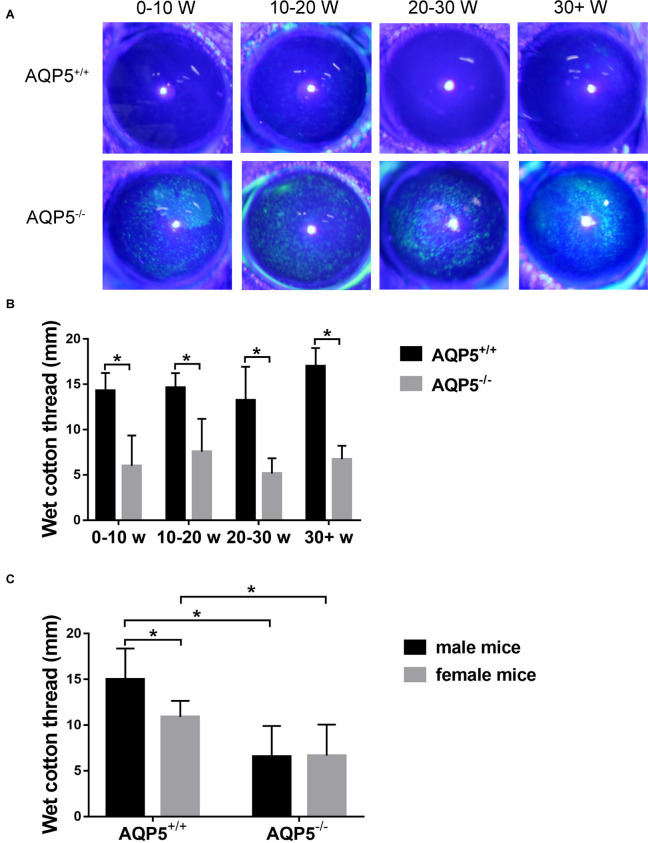
Effect of AQP5 knockout on the tear secretion of mice. **(A)** The results of sodium fluorescein staining in the cornea of AQP5^+/+^ mice and AQP5^–/–^ mice at different ages. The staining point was a corneal defect. **(B,C)** The lacrimal secretion of AQP5^+/+^ mice and AQP5^–/–^ mice was measured using the phenol red cotton thread method. The wet length of phenol red cotton thread in mice of different ages **(B)** and genders **(C)**. The data are expressed as mean ± *SD* (*n* = 14 per group). *p < 0.05.

In the course of our routine breeding of AQP5^–/–^ mice, we found that these mice exhibited spontaneous dry eye symptoms. Under a slit lamp, a spontaneous punctate epithelial defect in the cornea of these mice could be observed (Figure 1A). Then, we used a phenol red cotton thread to measure tears in AQP5^–/–^ mice by using AQP5^+/+^ mice as control. The tear volume of AQP5^–/–^ mice was significantly lesser than that of AQP5^+/+^ mice (Figure 1B). However, there was no difference between the tear volume produced by male and female AQP5^–/–^ mice (Figure 1C).

### AQP5 Deficiency Changed the Structure of Epithelial Cells in Lacrimal Glands

**FIGURE 2:**
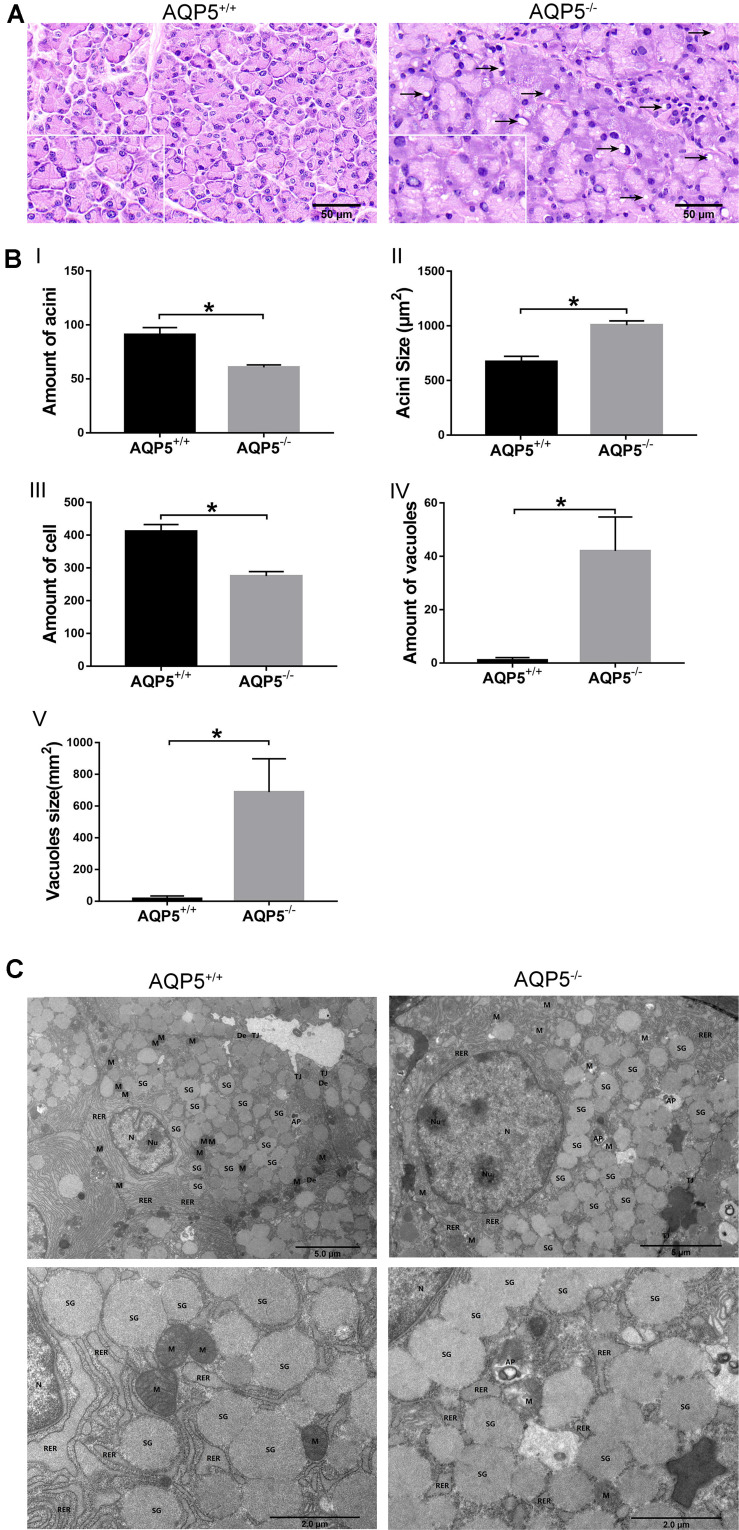
Structural changes of lacrimal glands in AQP5^–/–^ mice compared to AQP5^+/+^ mice. **(A)** Hematoxylin–eosin staining of lacrimal glands from AQP5^+/+^ mice and AQP5^–/–^ mice. Arrows indicate vacuoles inside the cytoplasm of AQP5^–/–^ acinar cells. **(B)** The number (I) and the area of single acini (II) in the unit area, the number of lacrimal gland epithelial cells in the unit area (III), and the number (IV) and area of vacuoles (V) in the unit area were compared between AQP5^–/–^ and AQP5^+/+^mice. The data are expressed as mean ± *SD* (*n* = 6 per group). *p < 0.05. **(C)** Transmission electron microscopy of lacrimal glands from AQP5^+/+^ mice and AQP5^–/–^ mice. N, nuclei; Nu, nucleolus; M, mitochondrion; RER, rough endoplasmic reticulum; SG, secretory granule; TJ, tight junction; DE, desmosome; AP, autophagosome; triangles, intercellular space.

Hematoxylin–eosin staining revealed that there were more vacuoles in the epithelial cells of the lacrimal glands of AQP5^–/–^ mice than in those of AQP5^+/+^ mice, and the arrangement of the acini was disordered in AQP5^–/–^ mice than in AQP5^+/+^ mice (Figure 2A). The specific observations in AQP5^–/–^ mice were as follows: the number of acini decreased (Figure 2BI), the area of single acinus increased (Figure 2BII), and the number of epithelial cells decreased (Figure 2BIII), and at the same time, the number and the proportion of vacuoles in lacrimal gland epithelial cells per unit area increased significantly (Figures 2BIV,V).

To further study the structural changes in the epithelial cells of lacrimal glands, the lacrimal glands of both groups of mice were subjected to electron microscopy analysis. The results showed that compared with the epithelial cells of AQP5^+/+^ mice, those of AQP5^–/–^ mice were seriously damaged, the mitochondria were swollen and enlarged, a large number of mitochondrial cristae had disappeared, the membrane had disintegrated, some secretory granules were dissolved, the secretory granules were fused, the levels of autophagy bodies increased, the cell gap was enlarged, and the endoplasmic reticulum around the cell membrane had expanded (Figure 2C).

### Overview of CircRNA Expression in Lacrimal Glands

**FIGURE 3:**
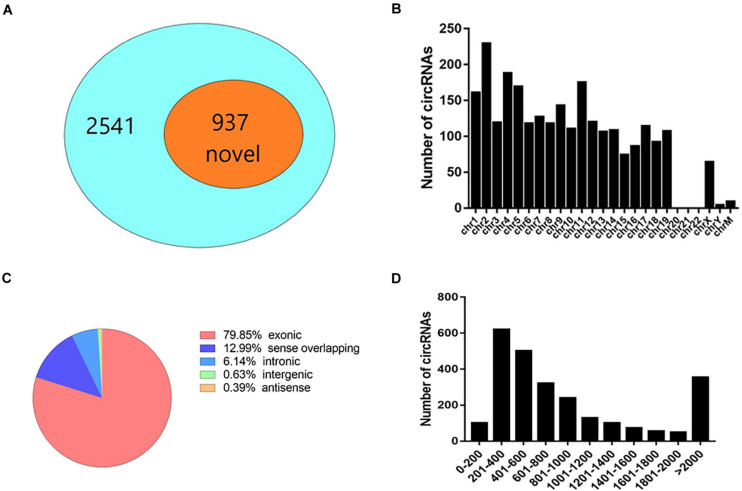
Expression pattern of circRNA in the lacrimal glands of C57BL/6 mice. **(A)** The proportion of newly discovered circRNA in all identified circRNA. **(B)** Distribution of circRNA on chromosomes. **(C)** The genomic origin of detected circRNA. **(D)** The length distribution of exonic circRNA.

To study the effect of AQP5 deficiency on lacrimal gland performance, we obtained lacrimal glands from AQP5^–/–^ and AQP5^+/+^ mice and sequenced their RNA with high throughput to determine the expression profile of circRNA in the glands. A total of 2,541 circRNAs were identified in the lacrimal glands of the two groups, 937 of which have not been reported before (Figure 3A).

Most circRNAs were located on chromosomes 1–19 (Figure 3B). There was a variety of catalogs of circRNAs, and most of them were exonic circRNAs (Figure 3C). The size of these circRNAs ranged from 82 nucleotides (nt) to more than 10,000 nt (Figure 3D). The overall average length was 2,824 nt.

### Differential Expression of CircRNAs in the Lacrimal Glands of AQP5^–/–^ and AQP5^+/+^ Mice

**FIGURE 4:**
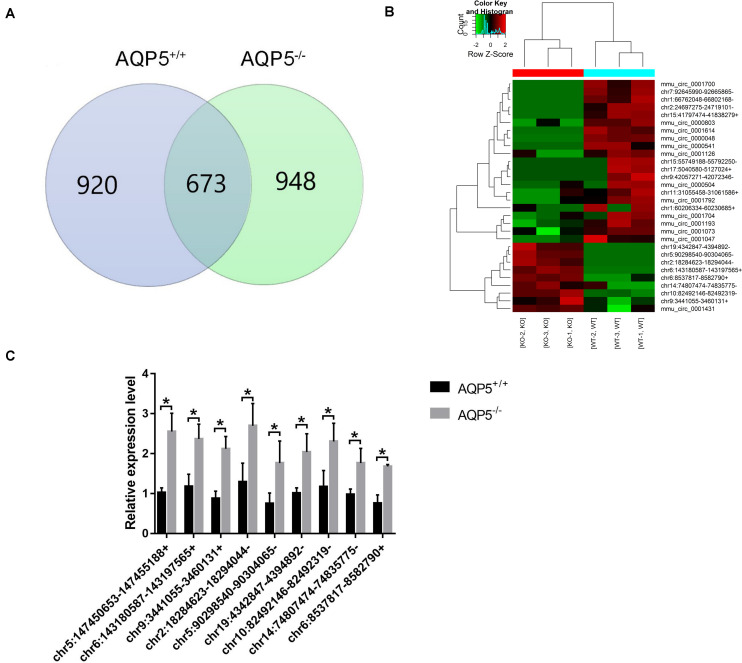
Identification of differential expression circRNA after AQP5 knockout. **(A)** Venn diagram of differentially expressed circRNA; 673 circRNAs were detected in both mice. **(B)** Clustering graph. Hierarchical clustering displayed the circRNA expression profile of AQP5^+/+^ mice (*n* = 3) vs. AQP5^+/+^ mice (*n* = 3). **(C)** The expression of nine upregulated circRNAs in AQP5^+/+^ and AQP5^+/+^ mice was detected by real-time fluorescence quantitative PCR. *p < 0.05.

Among the 2,541 identified circRNAs, 920 could only be detected in AQP5^+/+^ mice, 948 could only be detected in AQP5^–/–^ mice, and 673 could be detected in both types of mice (Figure 4A). Thirty circRNAs were differentially expressed in AQP5^–/–^ mice compared to AQP5^+/+^ mice, wherein nine of them were upregulated, while 21 were downregulated. Hierarchical clustering showed a distinguishable circRNA expression profile between AQP5^–/–^ and AQP5^+/+^ mice (Figure 4B).

To verify the high-throughput sequencing results, quantitative real-time polymerase chain reaction was performed for the nine upregulated circRNAs. The relative expression level of the nine circRNAs was consistent with that determined by high-throughput sequencing (Figure 4C).

### Function of Differentially Expressed CircRNAs in the Lacrimal Glands of AQP5^–/–^ and AQP5^+/+^ Mice

**FIGURE 5:**
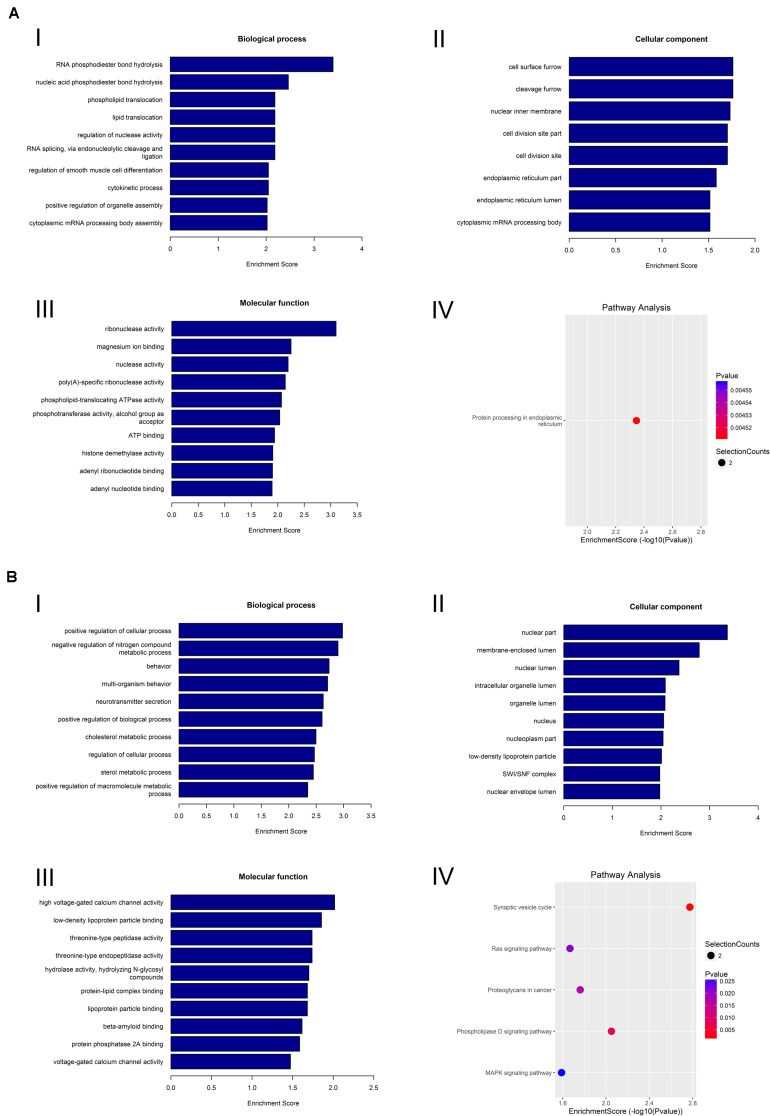
GO analysis and KEGG analysis of circRNA. **(A)** GO analysis and KEGG analysis of upregulated circRNA. GO analysis identified (I) molecular functions, (II) biological processes, and (III) cellular components. (IV) Relevant pathways were identified for upregulated circRNA. **(B)** GO analysis and KEGG pathway analysis of downregulated circRNA. GO analysis identified (I) molecular functions, (II) biological processes, and (III) cellular components. (IV) Relevant pathways were identified for downregulated circRNA.

To preferably understand the biological function of the differentially expressed circRNAs present in the lacrimal glands of AQP5^–/–^ mice, we performed Gene Ontology (GO) and Kyoto Encyclopedia of Genes and Genomes (KEGG) pathway enrichment analyses. The GO terms mainly covered three areas: biological processes, cellular components, and molecular functions. We found that the most abundant GO terms in the upregulated circRNAs were RNA phosphodiester in the endoplasmic reticulum, cell surface furrow, and ribonuclease activity. According to the KEGG analysis, the pathway related to the upregulation of circRNAs was protein processing in the endoplasmic reticulum (Figure 5A).

In the downregulated circRNAs, the most abundant GO terms were positive regulation of cellular process, nuclear part, and high-voltage-gated calcium channel activity. The most relevant pathway of these circRNAs was the synaptic vesicle cycle (Figure 5B).

### Differential Expression of mRNAs in the Lacrimal Glands of AQP5^–/–^ and AQP5^+/+^ Mice

**FIGURE 6:**
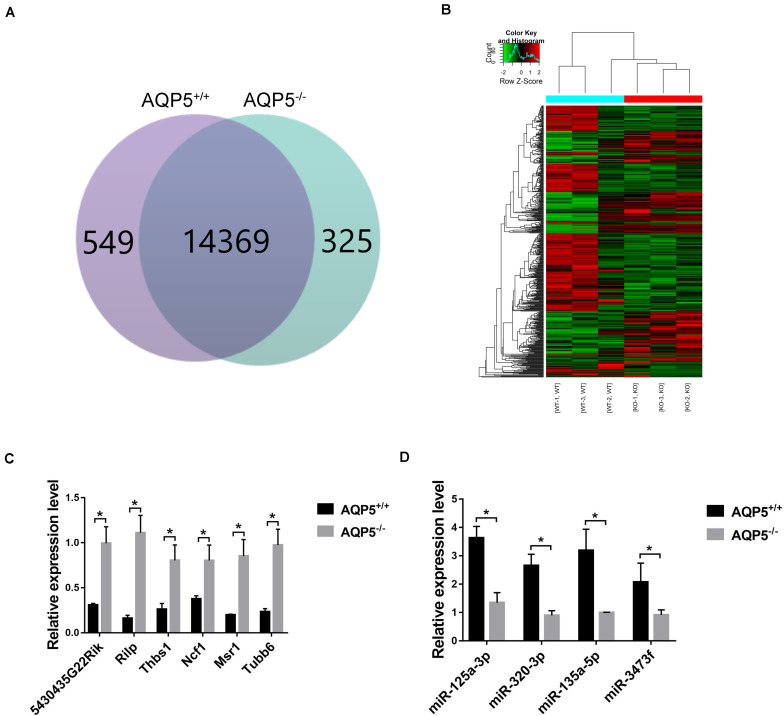
Identification of differential expression of mRNA after AQP5 knockout. **(A)** Venn diagram of differentially expressed mRNA; 15,243 mRNAs were detected in both mice. **(B)** Clustering graph. Hierarchical clustering displayed the mRNA expression profile of the AQP5^–/–^ mice (*n* = 3) vs. the AQP5^+/+^ mice (*n* = 3). **(C)** The expression of six upregulated mRNAs in AQP5^+/+^ and AQP5^+/+^ mice was detected by real-time fluorescence quantitative PCR. **(D)** The expression of four downregulated miRNAs in AQP5^+/+^ and AQP5^+/+^ mice was detected by real-time fluorescence quantitative PCR. *p < 0.05.

By high-throughput analysis, 15,243 messenger RNAs (mRNAs) were detected in the lacrimal glands of both AQP5^–/–^ and AQP5^+/+^ mice. Among these mRNAs, 549 could only be detected in AQP5^+/+^ mice, 325 could only be detected in AQP5^–/–^ mice, and 14,369 could be detected in both types of mice (Figure 6A). Compared to the mRNAs found in AQP5^+/+^ mice, 515 mRNAs were differentially expressed in AQP5^–/–^ mice, wherein 229 were upregulated and 286 were downregulated. Hierarchical clustering showed a distinguishable mRNA expression profile between AQP5^–/–^ and AQP5^+/+^ mice (Figure 6B).

To verify the high-throughput sequencing results, qRT-PCR was performed for the six upregulated mRNAs and four downregulated miRNAs. The relative expression levels of the six mRNAs (Figure 6C) and the four miRNAs (Figure 6D) were consistent with the sequencing results.

### Function of Differentially Expressed mRNAs in the Lacrimal Glands of AQP5^–/–^ and AQP5^+/+^ Mice

**FIGURE 7:**
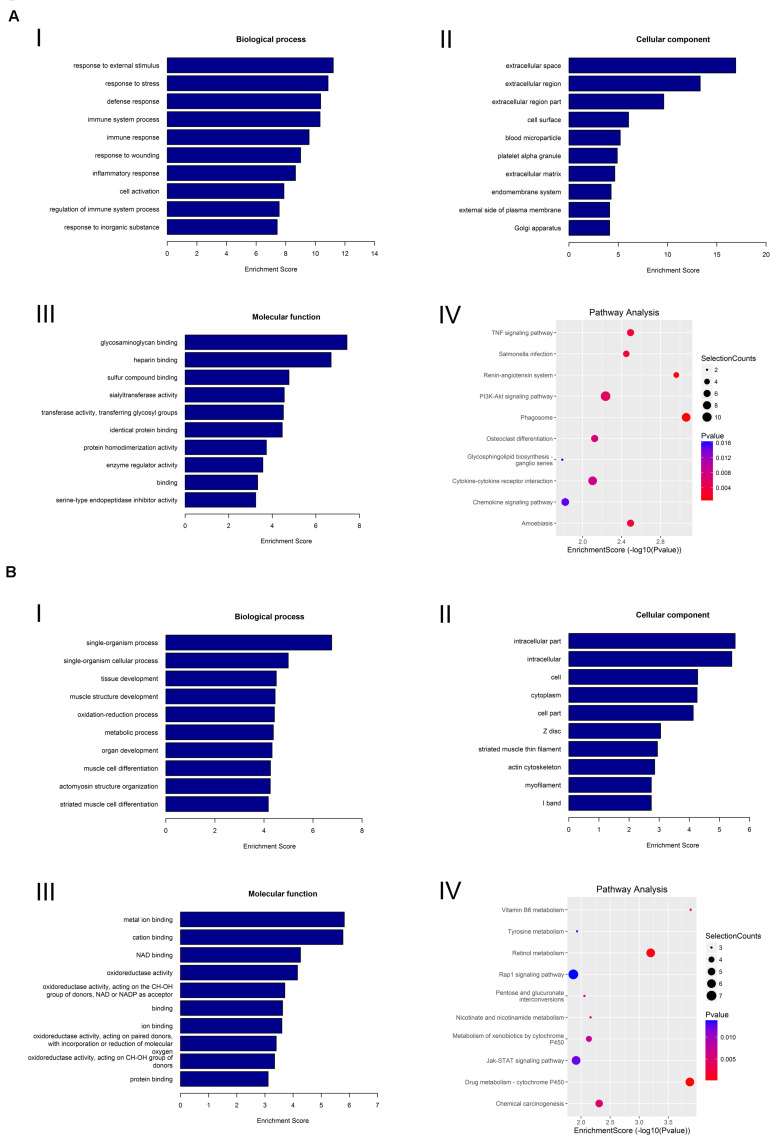
GO analysis and KEGG pathway analysis of mRNA. **(A)** GO analysis and KEGG pathway analysis of upregulated mRNA. GO analysis identified (I) molecular functions, (II) biological processes, and (III) cellular components. (IV) Relevant pathways were identified for upregulated mRNA. **(B)** GO analysis and KEGG pathway analysis of downregulated mRNA. GO identified (I) molecular functions, (II) biological processes, and (III) cellular components. (IV) Relevant pathways were identified for downregulated mRNA.

We also performed GO and KEGG pathway analyses on the differentially expressed mRNAs to determine their biological function in the lacrimal glands of AQP5^–/–^ mice. We found that the most abundant GO terms for the upregulated mRNAs were response to external stimulus, extracellular space, and glycosaminoglycan binding. According to the KEGG analysis, the pathway related to circRNA upregulation was phagosome (Figure 7A).

For the downregulated mRNAs, the most abundant GO terms were single-organism process, intracellular part, and metal ion binding. The most relevant pathway pertaining to the downregulated mRNAs was vitamin B6 metabolism (Figure 7B).

### CircRNA–miRNA–mRNA Network Analysis

To further determine the mechanism(s) underlying the functions of the identified circRNAs, we constructed a ceRNA regulatory network of circRNA–miRNA–mRNA. We targeted miRNAs and mRNAs using miRNA target prediction software according to TargetScan and miRanda. The top five miRNAs associated with each circRNA were involved in the network. All the targeted mRNAs of each of the top five miRNAs matched with the upregulated mRNAs that were identified by mRNA sequencing.

**FIGURE 8:**
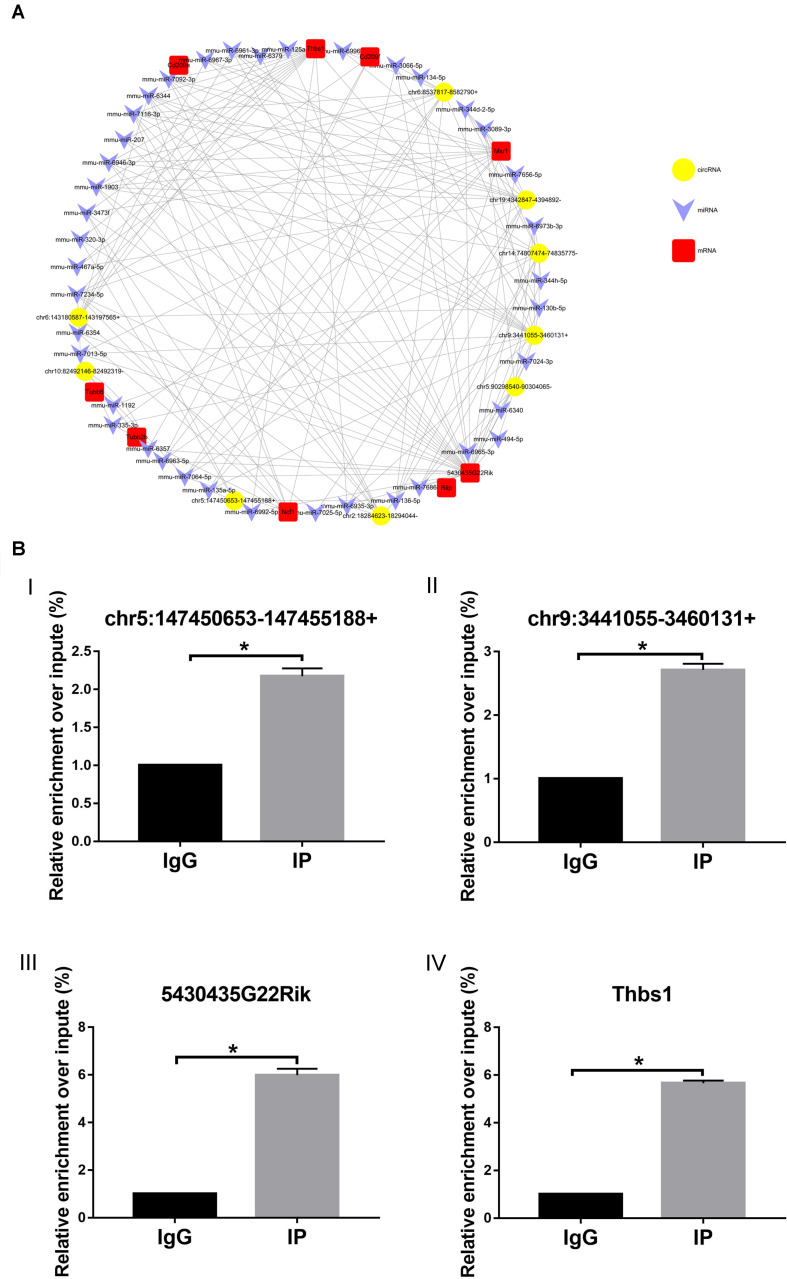
circRNA–miRNA–mRNA network. **(A)** Circles represent circRNA, arrowhead represents miRNA, and squares represent mRNA. **(B)** RIP-qPCR detected (I) chr5:147450653-147455188+, (II) chr9:3441055-3460131+, (III) 5430435G22Rik, and (IV) Thbs1. IG, negative control immunoglobulin G group; IP, containing magnetic beads of anti-AGO2 antibody group. *p < 0.05.

The network was constructed by using nine upregulated validated circRNAs, 40 altered miRNAs, and nine upregulated mRNAs (Figure 8). In this complex network, one circRNA can associate with multiple miRNAs and one miRNA can regulate multiple mRNAs. According to the comparison with the database, these nine mRNAs and phagosome pathways are also closely related.

To further study the function of the previously identified circRNAs and verify the reliability of the predicted network of circRNA–miRNA–mRNA, RIP-qPCR was performed. As shown in Figure 8, compared with the IgG group (control), chr5:147450653-147455188 + (Figure 8BI) and chr9:3441055-3460131 + (Figure 8BII) can combine with the AGO2–miRNA complex, suggesting that chr5:147450653-147455188 + and chr9:3441055-3460131 + can combine with miRNA through a sponge mechanism. In addition, compared with the IgG group (control), 5430435G22Rik (Figure 8BIII) and Thbs1 (Figure 8BIV), identified in the previously predicted network graph, could also be detected, which indicates that our prediction is reliable.

## Discussion

Dry eye is a common chronic ocular surface disease caused by many factors such as reduced tear film stability, increase in tear osmolality, and ocular surface inflammation (Nelson et al., 2017). With changes in people’s working environments and living habits, the incidence of dry eye is increasing annually, but there are few effective drugs to treat it. This lack of effective drugs is mainly related to the diverse physiological symptoms associated with dry eye in humans and the lack of corresponding animal models for studying these diverse symptoms in detail.

Previous studies have used animal models of dry eye by administering drugs to inhibit the secretion of tears or by removing the lacrimal or Harderian glands of mice (Gilbard et al., 1987; Viau et al., 2008; Fakih et al., 2019). However, these methods are too complex and cumbersome, the efficiency of the models is not stable, and the damage to the mice is greater. Therefore, selecting a simpler and more stable animal model of dry eye for studying its pathogenesis and for treatment drug screening is particularly important. In this study, we found that AQP5^–/–^ mice naturally develop dry eye symptoms from birth, which makes them a stable, simple, and effective dry eye research animal model that can be used to study the pathogenesis of this condition. The AQP5 level has been considered to decrease with the occurrence of dry eye and is usually regarded as an indicator of dry eye in fundamental research (Wang et al., 2017). However, whether dry eye occurs because of *AQP5* knockout has not been reported.

The occurrence of dry eye is usually related to age and gender; the incidence gradually increases with age, and women are more likely to develop dry eye than men (Kaštelan et al., 2013). However, in this study, we did not observe this phenomenon. AQP5^–/–^ mice had dry eyes since birth, and no age- and gender-related trends were observed. This may be because the type of dry eye after AQP5 knockout is primary.

H*e*matoxylin–eosin staining of the lacrimal gland specimens of AQP5^–/–^ mice revealed that the structure of the acini was disordered, and there were many changes in the vacuolation structure in the acini. The same phenomenon was observed in TSP^–/–^ mice, a mouse model of Sjögren’s syndrome (an autoimmune disease) (Bhattacharya et al., 2018). Electron microscopic observations revealed that a large number of mitochondrial cristae had disappeared, and there was an increase in the number of autophagosomes. These results suggest that the structural changes in the lacrimal gland epithelial cells of AQP5^–/–^ mice may be related to autophagy.

As new non-coding RNAs, circRNAs play a key role in many ophthalmic diseases such as macular degeneration and corneal neovascularization (Liu et al., 2020; Wu et al., 2020). However, the role of circRNAs in dry eye and lacrimal gland development has not been studied before. Thus, to better understand the complex pathogenesis of dry eye, identification of comprehensive circRNA expression profiles in the lacrimal glands of AQP5^–/–^ mice was critical. We identified 2,541 circRNAs, of which 937 were newly identified. We also identified 30 differentially expressed circRNAs in the lacrimal glands of AQP5^–/–^ mice compared with those of control mice. We selected nine upregulated circRNAs for validation. The qRT-PCR results were consistent with those of high-throughput sequencing, which proved the reliability of high-throughput sequencing.

In the circRNA–miRN–mRNA regulatory network or the ceRNA theory, circRNAs play the role of miRNA sponge and influence the expression of target mRNAs by competitive adsorption of miRNAs (Qi et al., 2015). We found the circRNA and the mRNA with the same trend by sequencing circRNA and mRNA and then find the circRNA and mRNA with the same miRNA binding site by bioinformatics analysis. Only by meeting the above two conditions can the circRNA and mRNA be used to construct the circRNA–miRNA–mRNA regulatory network. Based on the nine validated upregulated circRNAs and the top five miRNAs predicted by using circRNAs, we predicted a circRNA–miRNA–mRNA network. In addition, by performing RIP-qPCR experiments, we proved that our predicted network is reliable. As a known member of the Argonaute protein family, Argonaute 2 (AGO2) can combine with miRNA to form the AGO2–miRNA complex (Zhang et al., 2020). This complex can be combined with circRNA *via* the sponge mechanism, which proves that chr5:147450653-147455188 + and chr9:3441055-3460131 + can play a role through sponge mechanism. In addition, the circRNAs and the mRNAs identified in our previously predicted network can also be detected by combining with the AGO2–miRNA complex, which shows that our network is reliable.

Interestingly, the target genes involved in this network are all related to phagosomes, according to the comparison with the database. These observations highly correlated with the results of our KEGG analysis of upregulated mRNA. Phagosomes, as a highly dynamic organelle, play an important role in innate and adaptive immunity as well as in tissue homeostasis (Dean et al., 2019). Our results indicate that the circRNA–miRNA–mRNA network predicted in our study is a network of phagosomes. What is more surprising is that our prediction is consistent with the structural changes in the lacrimal gland epithelial cells that we mentioned before. Therefore, these circRNAs may be involved in the regulatory networks and the pathways of phagosomes involved in the pathogenesis of dry eye. However, the regulatory mechanism of circRNAs is very complex; hence, it is necessary to further study the function and the mechanism of these circRNAs.

## Conclusion

The present study demonstrates that (i) *AQP5* knockout can lead to primary dry eye development in mice (AQP5^–/–^ mice have a stable dry eye phenotype since birth) and this phenotype may be produced by altering the structure of lacrimal glands, (ii) circRNA levels are significantly altered in the lacrimal glands of AQP5^–/–^ mice, and (iii) the interactions of the circRNA–miRNA–mRNA network associated with phagosomes may regulate the expression of *AQP5* involved in the pathogenesis of dry eye.

## Data Availability Statement

The data of sequencing results for this study can be found in the GEO database (GSE149832). Please see https://www.ncbi.nlm.nih.gov/geo/query/acc.cgi?acc=GSE149832 for more details.

## Ethics Statement

All experimental and animal care procedures followed the ARVO Statement for the Use of Animals in Ophthalmic and Vision Research and were approved by the Animal Care and Use Committee of Qingdao University (Qingdao, China).

## Author Contributions

GD and PC conceived and designed the study. YL, SH, XX, and XW performed the experiments. GD and TZ analyzed the data. YL and PC wrote the manuscript. All authors read and approved the final manuscript.

## Conflict of Interest

The authors declare that the research was conducted in the absence of any commercial or financial relationships that could be construed as a potential conflict of interest.

## Abbreviations

AGO2: argonauute 2
AQP5: aquaporin 5
circRNA: circular RNA
GAPDH: glyceraldehyde 3-phosphate dehydrogenase
GO: Gene Ontology
KEGG: Kyoto Encyclopedia of Genes and Genomes
miRNA: microRNA
mRNA: messenger RNA
nt: nucleotides
qRT-PCR: quantitative real-time polymerase chain reaction
rRNAs: ribosomal RNA
RIP: RNA immunoprecipitation
SD: standard deviation.
